# Unexpectedly Low Rangewide Population Genetic Structure of the Imperiled Eastern Box Turtle *Terrapene c. carolina*

**DOI:** 10.1371/journal.pone.0092274

**Published:** 2014-03-19

**Authors:** Steven J. A. Kimble, O. E. Rhodes Jr., Rod N. Williams

**Affiliations:** 1 Department of Forestry and Natural Resources, Purdue University, West Lafayette, Indiana, United States of America; 2 Savannah River Ecology Laboratory, Aiken, South Carolina, United States of America; Macquarie University, Australia

## Abstract

Rangewide studies of genetic parameters can elucidate patterns and processes that operate only over large geographic scales. Herein, we present a rangewide population genetic assessment of the eastern box turtle *Terrapene c. carolina*, a species that is in steep decline across its range. To inform conservation planning for this species, we address the hypothesis that disruptions to demographic and movement parameters associated with the decline of the eastern box turtle has resulted in distinctive genetic signatures in the form of low genetic diversity, high population structuring, and decreased gene flow. We used microsatellite genotype data from (n = 799) individuals from across the species range to perform two Bayesian population assignment approaches, two methods for comparing historical and contemporary migration among populations, an evaluation of isolation by distance, and a method for detecting barriers to gene flow. Both Bayesian methods of population assignment indicated that there are two populations rangewide, both of which have maintained high levels of genetic diversity (*H_O_* = 0.756). Evidence of isolation by distance was detected in this species at a spatial scale of 300 – 500 km, and the Appalachian Mountains were identified as the primary barrier to gene flow across the species range. We also found evidence for historical but not contemporary migration between populations. Our prediction of many, highly structured populations across the range was not supported. This may point to cryptic contemporary gene flow, which might in turn be explained by the presence of rare transients in populations. However these data may be influenced by historical signatures of genetic connectivity because individuals of this species can be long-lived.

## Introduction

The quantification of genetic parameters of species is basic to understanding their natural history. This is especially important in declining species in need of appropriate conservation approaches [1]. Species in decline, however, have by definition undergone demographic reductions [2], which may confound our ability to differentiate between natural and anthropogenically induced changes in genetic parameters. For example, when studying long-lived species investigators must distinguish between pre-disturbance (and therefore presumably stable) and anthropogenically induced genetic patterns to inform management strategies [3], [4]. Furthermore, population genetic studies involving declining species are often confined to drawing broad management conclusions from limited data on a few individuals or populations, resulting in deviations from analytical assumptions which can negatively affect the reliability of results [5].

Wide-ranging species of conservation concern, whose interpopulation migration patterns are influenced by habitat fragmentation, also could be expected to exhibit genetic evidence of disruption to large- and small-scale movement behaviors [6]. Such species are often characterized by high dispersal (intrapopulation movement) and migration (interpopulation movement) habits, and thus are expected to be vulnerable to habitat fragmentation [7], [8]. Unfortunately, the genetic attributes of many declining, wide-ranging species are poorly studied over large spatial scales, despite the fact that many are marked for conservation management planning.

A genetic population is commonly defined in the field as a group of conspecifics that are genetically similar and are to varying degrees separated from other populations of conspecifics and are likely to be more locally adapted [9]. Populations of long-lived, wide-ranging species should be managed at geographic scales that are appropriate for conservation planning at multiple levels of biological resolution [10], [11]. Management plans conducted at geographic scales significantly smaller than that of the population may fail to incorporate mechanisms that maintain genetic diversity [12], while those conducted at scales larger than that of the population may lead to the loss of locally adapted genes [13]. For example, anthropogenic interpopulation movement of genotypes may introduce alleles that are locally maladaptive in the receiving population [13], [14]. In addition, rangewide approaches are tremendously useful for identification of significant management units, i.e., genetic populations for which management plans should be made for species or populations of conservation concern [1], [15] even those with low population differentiation [11]. Determination of the appropriate scale for management of turtles, in particular, is of paramount importance [16] because approximately 40% of Chelonians worldwide are considered endangered or vulnerable [16], [17]. Simply removing local sources of extrinsic stressors, without regard to range-wide reservoirs of genetic resources, may not be sufficient mitigation against loss of genetic diversity because future threats such as climate change and novel disease may overwhelm genetically depauperate species [4], [16].

The eastern box turtle *Terrapene c. carolina* is a declining species whose conservation plans are difficult to develop because of the complexities of studying a species that is cryptic, long-lived (>100 years in the wild; N. Karraker, unpublished data; [18]), and whose generations overlap. It is a terrestrial turtle species that historically ranged across much of the eastern United States [19], but which has suffered substantial demographic declines [19]–[22], likely due to some combination of habitat destruction and fragmentation, road mortality, collection, and disease [19]. Understanding the effects of demographic declines on patterns of genetic diversity and structure in box turtles is a necessary precursor to developing management strategies for their protection, but previous studies suggest that such studies should be conducted at scales larger than single states [23]–[25].

Our intent here was to explore range-wide genetic patterns of the eastern box turtle as a means to inform conservation planning for this species and provide a model for other species with similar traits and demographic histories. To accomplish this we first tested the hypothesis that genetic isolation by distance is significant across the species range due to the limited migration ability of this species. Second, we tested the hypothesis that habitat loss and fragmentation has formed multiple, geographically discrete and genetically differentiated populations across the species range. Because habitat reduction increases distance among patches of suitable habitat, we also tested a third hypothesis that increasing isolation due to habitat fragmentation has caused a reduction in the number of migrants among populations.

## Methods

### Sample collection

**Figure 1:**
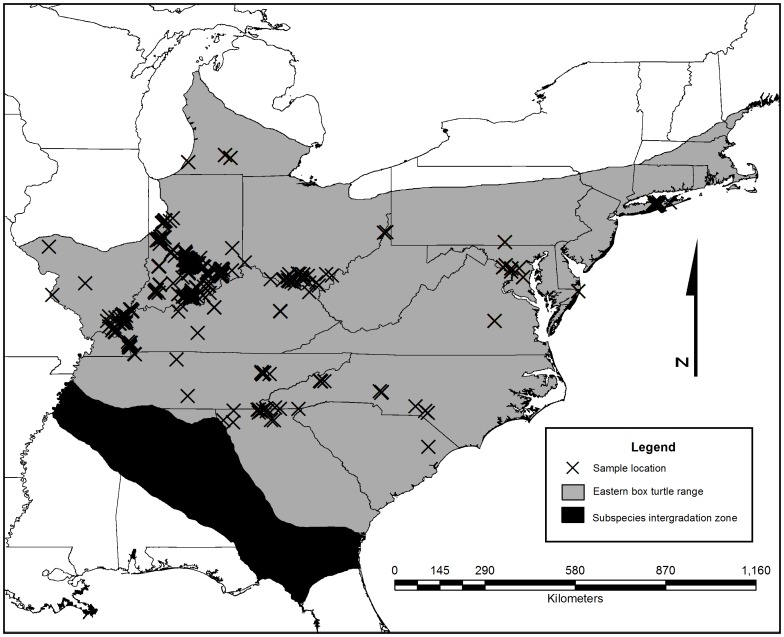
Locations of rangewide samples collected from *Terrapene c. carolina*. Due to the resolution, some marks represent more than one sample. Range data after Dodd (2001).

We conducted searches for eastern box turtles via visual encounter by car and on foot across much of their range (Figure 1). We sampled at multiple geographic scales to determine at what spatial extent populations occur. To this end, we sampled at eight sites in Indiana (a state whose forests are heavily fragmented by agriculture; [26]), in the four states surrounding Indiana (Illinois, Kentucky, Ohio, and Michigan), and from another nine states across the species range. We intentionally avoided sampling in the south and southwestern parts of the range where the eastern box turtle is sympatric with two other subspecies of *T. carolina* [25] to avoid confounding results with alleles from different subspecies. For each individual we recorded UTM coordinates, morphometric data, sex, activity, and any unusual markings or signs of injury or disease. We took tissue samples for genetic analysis, usually blood (∼10μL), following the protocol of Kimble and Williams [27]. We assigned each turtle a unique number and filed a corresponding pattern of notches into the marginal scutes [28] to identify recaptures. These notches were subsequently sealed with surgical adhesive. Individuals were processed as quickly as possible and released immediately at the point of capture.

All animals we handled were done so in accordance with the Purdue Animal Care and Use Protocol 07-037 and amendments thereto. For all animals we handled we obtained all relevant permissions and permits from the appropriate government agencies, land trusts, and property managers before sampling began. We sampled in the Chattahoochee National Forest area of Georgia under permits from the USDA Forest Service Chattahoochee-Oconee National Forest office and Georgia Department of Natural Resources, Wildlife Resources Division; in Illinois under a permit from the Illinois Department of Natural Resources; in Indiana under permits from the Indiana Department of Natural Resources, Fish and Wildlife Division and Division of Nature Preserves and under permissions from NICHES Land Trust and Wabash College; in New York under a permit from the New York Department of Environmental Conservation, Division of Fish, Wildlife and Marine Resources; in North Carolina under a permit from the North Carolina Wildlife Resources Commission, Division of Wildlife Management; and in Ohio under the Ohio Department of Natural Resources, Division of Wildlife. Samples from other states were collected by collaborators working under their own permits.

### Laboratory

We digested tissue samples using a modified proteinase K protocol and extracted DNA with a phenol-chloroform-isoamyl alcohol [29]. We resuspended purified DNA in 50 μL of TLE (10 mM tris, 0.1 mM EDTA, pH 8.0) and quantified DNA concentration on a spectrophotometer (NanoDrop 8000, Thermo Scientific, Wilmington, DE). We then diluted all DNA samples to 20 ng/μL in pure water prior to PCR.

We carried out PCR using 11 microsatellite loci developed specifically for the eastern box turtle [30]. We combined the 11 loci into three multiplexes and two singletons (Table 1). All reactions contained 60 ng DNA template, 10 mM tris-HCl, 0.05 mg/mL BSA, 50 mM KCl, 0.9 mM MgCl_2_, 0.2 mM of each dNTP, 0.3 U of Taq polymerase, and multiplex-specific concentration of end-labeled fluorescent primers in a total reaction volume of 10 μL. We analyzed all PCR products on an automatic sequencer (ABI 3730XL, Applied Biosystems, Foster City, CA). We automatically scored genotypes with Genemapper (version 3.7, Applied Biosystems, Foster City, CA) and checked each call manually at least twice. We reamplified ∼10% of all genotypes to ensure repeatability and reamplified any that disagreed a third time. We used Cervus (version 3.0.3; [31]) to check for accidentally duplicated samples and in the case of duplication removed all but the first sample taken from an individual.

**Table 1:** Microsatellite PCR multiplex parameters for the eastern box turtle, *Terrapene. c. carolina*.

Multiplex	Locus	T_A_ (°C)	Primer concentration (μM)	Fluorophore
I	TCC_di_045	58.0	0.3	6-FAM
I	TCC_tetra_070	58.0	0.3	NED
I	TCC_di_082^1^	58.0	0.3	6-FAM
II	TCC_di_189	60.0	0.6	6-FAM
II	TCC_tetra_043	60.0	0.6	HEX
II	TCC_tetra_012/342	60.0	0.3	NED
III	TCC_di_366	63.0	0.5	6-FAM
III	TCC_di_318	63.0	0.3	HEX
III	TCC_di_352	63.0	0.3	NED
IV	TCC_di_345	63.0	0.3	6-FAM
V	TCC_di_300	57.0	0.3	6-FAM

1 TCC_di_082 was PCR amplified separately and pooled with Multiplex I before genotyping.

### Statistical analysis

To test for the presence of null alleles and large allelic dropout, we used Microchecker (version 2.2.3; [32]). To assess how well our dataset and each population met Hardy-Weinberg equilibrium (HWE) assumptions, we used the web version of Genepop (version 4.0.10; [33]) to test for a significant deficit of heterozygotes using an exact method [34] with default parameters. We used a Markov chain Monte Carlo (MCMC) method [35] to assess significant deviations from HWE. We quantified deviations from Hardy-Weinberg equilibrium (HWE) by estimating *F*_IS_ in Genepop with 10,000 iterations.

### Isolation by distance

We evaluated rangewide isolation by distance by using a Mantel test [36] performed in Alleles in Space (Ais, version 1.0; [37]) to determine whether a significant correlation existed between pairwise matrices of Nei's [38] genetic distances and geographic distances matrices. We log_10_-transformed the geographic distance matrix to meet the assumption of normality [39] and used 1,000 randomized replicates to assess significance. To estimate the geographic scale at which IBD may begin to operate, we also performed a complementary spatial autocorrelation analysis in Ais, using 10 classes of equal distance and 1,000 replicates to assess significance.

### Determination of population number, individual assignments, and barriers

To improve analytical robustness against violations of software-specific assumptions, a common approach in population genetics [40], we used two Bayesian analyses to infer the size, shape, and individual membership of populations. The first approach was in Geneland (version 4.0.0; [41]) in program R [42] without spatial priors. We set Geneland to search for the most likely number of populations (*k*) from 1–25 over 1,000,000 MCMC iterations with thinning every 100^th^ iteration and a burn-in of 50,000. We assumed uncorrelated allele frequencies to avoid artifacts of uneven geographic sampling [43] and the potential for overestimation of *k* associated with the use of correlated frequency allele models [44]. We set the Poisson process maximum to 800 and the maximum number of nuclei allowed for the Poisson-Voronoi tessellation to 2,400 [41], [45]. We ran the MCMC 10 times for each value of *k* and used the highest mean probability density value as the inferred *k* value. We also estimated the probability of population assignment for each individual.

To corroborate results, we used the Bayesian algorithm Structure (version 2.3; [46]). We performed 10 independent runs for each value of *k* from 1 to 25 for 1,000,000 iterations, including a 50,000 iteration burn-in. We ran the model with default settings except we used an admixture model, and with allele frequencies uncorrelated (as with Geneland). To visualize and infer the most likely value of *k* we used Structure Harvester (version 0.6.92; [47]), which employs the Δ*k* method of Evanno et al [48]. Structure also assigns a probability value to each the population assignment of each individual. For both Geneland and Structure we analyzed each population individually for substructuring. We compared the results of both algorithms and used the population assignment with the highest confidence per individual for further analyses. Finally, we compared the individuals assigned to each population by both Geneland and Structure for agreement. We tested resulting genetic populations for violations of HWE and dropped loci with relatively high null allele estimates as necessary.

To estimate the location of specific natural or anthropogenic barriers to gene flow which might have contributed to population structure identified across the species range, we used the maximum difference algorithm of Monmonier [49], which has recently been applied to landscape genetics [45], [50]–[52]. This process builds a connectivity network of the sample locations using Delaunay triangulation, and then estimates the barriers among them by following contiguous connectivity links between samples that represent the highest genetic distances. We used this method in Ais using genetic distances corrected for geography (“pseudoslopes”). We set the number of barriers to *k* - 1, with *k* being the number of populations identified by Geneland and Structure.

Previous studies in the population genetics of *Terrapene* species suggest that populations may operate at geographic scales greater than most management jurisdictions, such as state or national parks, and perhaps even larger than a single state [4], [23], [25]. If this is the case, managers of local sites cannot manage for the entire population but instead must work with the genetic reality of the individuals under their control. To this end, we also report results for each management unit in which we sampled at least nine individuals (Table S1).

### Quantification of migration among populations

We used the genetic population clusters identified by the Bayesian analyses to detect recent migration among them using Bayesass (version 3; [53]), a genotype-based Bayesian platform appropriate even for populations that do not meet assumptions of HWE. We ran 1,000,000 iterations with a burn-in of 100,000 and sampling intervals of 100. We set the mixing parameters for the MCMC chain to 0.02 for migration, 0.1 for inbreeding, and 0.05 for allele frequencies so that the resulting migration parameter swapping acceptance rates were between 20% and 40% as suggested by the authors. We ran the analysis five times to check for convergence and visualized chain mixing, convergence, and burn-in values in Tracer (version 1.5; [54]).

We used Migrate (version 3.1.1; [55]) to estimate historical migration rates (among populations identified by the Bayesian clustering analyses). Under the maximum likelihood (ML) framework, we used a Brownian motion model of mutation, suitable for microsatellite data that likely do not adhere to a strictly stepwise model of mutation. We used five independent replicates of 10 short chains 10,000 iterations in length, three long chains 100,000 iterations in length, and four heated static chains at temperatures 1.0, 1.5, 3.0 and 10,000.

## Results

### Sample collection and laboratory

We collected tissue samples from 1,603 wild eastern box turtles from across much of the species range (Figure 1). We successfully resolved all quality control disagreements among PCR amplifications and between scorers and excluded all individuals with more than three loci for which genotype data could not be resolved (n = 45). Thus, the final data set included 1,558 individuals. For all genetic analyses, we randomly selected 24 individuals from the two locations where we sampled deeply for other purposes (the Hardwood Ecosystem Experiment in south-central Indiana: n = 627; and Oak Ridge, Tennessee: n = 182), to even the sampling distribution across sites. This resulted in a total of 799 individuals from which data were used for all analyses of genetic structure, gene flow and IBD.

### Statistical analysis

Mean allelic richness from the sample sites ranged from 7.6 to 33.6 (Table S1). When all 799 samples were pooled, there was a significant deviation from Hardy-Weinberg equilibrium across all 11 loci (χ^2^ = ∞, df = 22, *p*<0.001). Microchecker estimated the potential presence of numerous null alleles at low frequencies (Table S1). All but two of the management unit populations (e.g., Patuxent Wildlife Research Center in Maryland) were also significantly out of equilibrium, but these two have low sample sizes (9 and 12). A few loci suffered from heterozygote deficiencies in some populations (Table S2).

### Isolation by distance

**Figure 2:**
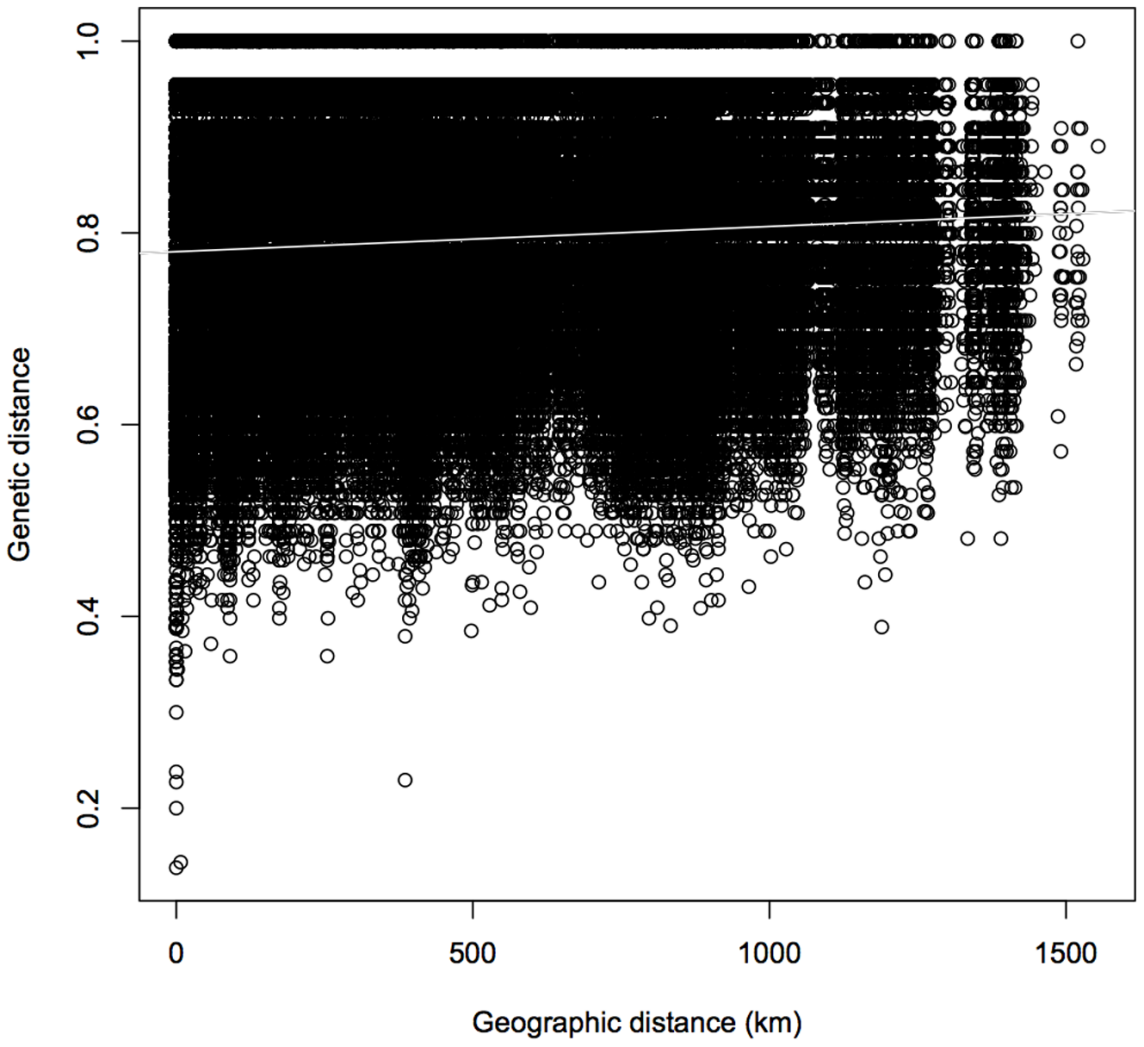
Correlogram of genetic isolation by geographic distance between pairs of eastern box turtle *Terrapene c. carolina* individuals across its range.

Isolation by distance was significant across the species range (Figure 2; *r* = 0.13, *p*<0.001). Additionally, the spatial autocorrelation analysis estimated that the pairwise genetic distance in the 300 – 500 km distance class (A_y_ = 0.795) exceeded the mean pairwise genetic distance for all pairwise distances (A_v_ = 0.793). This indicates that significant isolation begins to operate at this geographic distance and that panmixia operates at geographic scales less than this threshold [56], [57].

### Determination of population number, individual assignments, and barriers

**Figure 3:**
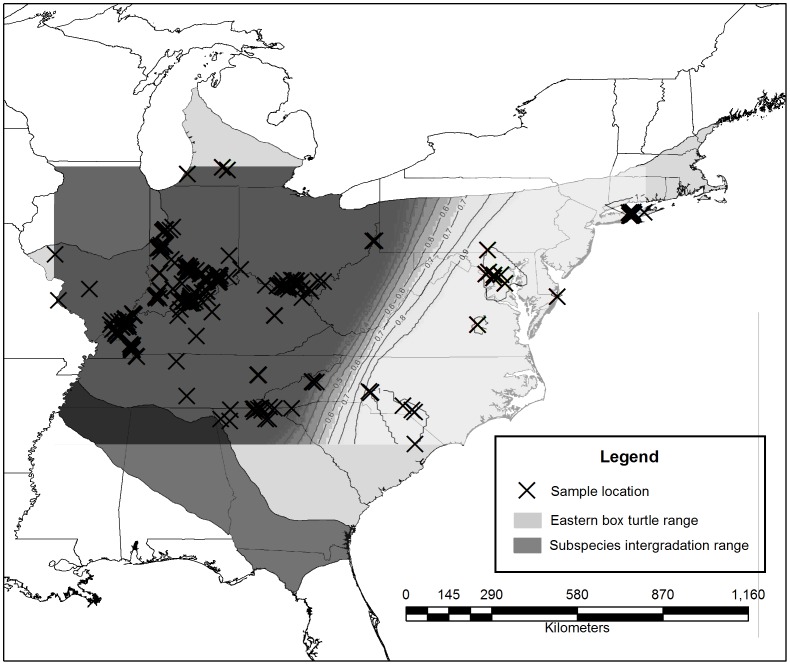
Map of the two populations found rangewide in the eastern box turtle *Terrapene. c. carolina*. The probability map of an individual turtle belonging to the Western population was generated by Geneland and increases with darker shades of gray. The probability of an individual belonging to the Eastern population would be proportionally opposite. Note that the border between the populations follows the Appalachian Mountains.

The nonspatial model Geneland reported *k* = 2 in 7 of 10 runs. One of the runs reporting *k* = 2 also had the highest mean of probability density value and so we used this as the point estimate of the number of populations. Secondary runs on each of the two clusters revealed no substantial substructuring, i.e., population assignment probabilities were low. The final map (Figure 3) shows the population boundary roughly following the spine of the Appalachian Mountains. The nonspatial model Structure also indicated that *k* = 2 according to the point value estimation method of Evanno et al [48]. More than 85% of individuals were assigned a population assignment probability of >0.9. Secondary analyses on these two clusters also showed no substantial substructuring, i.e., all Δ*k* values were low. Individuals with admixed ancestry did not tend to cluster along the boundary between the Western and Eastern populations, a signature of a zone of interbreeding. Structure and Geneland assigned 95.6% of individuals to the same two population groups.

There was a nearly universal lack of Hardy-Weinberg equilibrium, despite the fact that the Bayesian methods for population delineation construct populations by minimizing deviations from HWE and linkage. This might be caused by technical reasons (e.g., null alleles; [58]), or by the violation of HWE assumptions that are not appropriate for box turtles [9]). These deviations may be explained by the high polymorphisms at our loci, which range from 16 to 83 alleles per locus (mean: 36.1) and which resulted in the detection of many rare alleles. As allelic richness increases at a locus, the likelihood that they will be found as homozygotes declines in a finite sample size of the population, increasing the likelihood of deviations from HWE [9]. Furthermore, while H_E_ and H_O_ estimates vary by locus in each population, mean H_E_ and H_O_ estimates are similar across populations which suggests that departures from HWE are artifacts of the algorithms and do not compromise the interpretations of our data (Table S2). It has also been demonstrated that distinguishing between deviations from HWE and the presence of null alleles can be difficult, and the common tactic of excluding “problematic” loci may result in a loss of the most informative loci [59].

### Quantification of migration among populations

Because both Structure and Geneland were strongly concordant, we coded Ais to find one Monmonier barrier between them. The resulting line was largely congruent with the map that Geneland generated, drawing the barrier along a line running north to south from Pennsylvania through Maryland, Virginia, North Carolina and South Carolina. We refer to the two resulting populations hereafter as “Western” and “Eastern”. Though both Geneland and Structure operate by minimizing linkage disequilibrium and departures from HWE, both populations were also significantly out of HWE (Table S1). Both the Western and Eastern populations had three loci with a null allele rate 0.10 – 0.12 but exclusion of these from both the Bayesian approaches and the test for HWE had not substantial effect on the results.

Bayesass gave no evidence for recent immigration between populations as estimates included zero in their credible intervals. By contrast, Migrate estimated a historical migration rate of 15.2 migrants/generation from the Western population into the Eastern, and 17.4 migrants/generation from East to West.

## Discussion

Life history traits of *Terrapene* species make the formulation of clear hypotheses about the geographic patterns of genetic populations difficult. Much of the natural history evidence for *Terrapene* suggests that members of this genus should maintain population structure at relatively small geographic scales. For example, one *T. ornata* individual was recaptured 27 times over ten years within 7.6 m of its initial capture location [60] and home range size for *T. carolina* has been reported between 0.02 and 187.67 ha [19], [61]. Observational data suggest that juvenile dispersal may be very short distances, approximately 100 m or fewer [62], [63]. Ultimately, evidence of low dispersal and highly conserved adult home ranges suggests that members of this genus should display a high degree of population structure at small spatial scales.

Alternatively, there is some evidence to the contrary. First, the existence of transient adult box turtles has been hypothesized due to the lack of recapture, despite intense effort spanning decades [20], [21], [64]. Furthermore, individuals with transient behavior have been observed in two radiotelemetry studies (*T. c. triunguis*, [70]; S. Kimble, *T. c. carolina*, unpublished data.) where adult males traveled roughly linear paths that were many times longer than the width of the standard home range (∼10 km over two active seasons, [65]; ∼7 km over 1 active season, S. Kimble, unpublished data), and have been observed mating along the way [65]. Second, *T. carolina* box turtles are not closely tied to bodies of water [19], and thus may suffer from fewer geographic constraints on gene flow than do aquatic turtles. Third, while little is known about juvenile *Terrapene* dispersal [19], parent-offspring pairs have been found up to 27.1 km apart [S. Kimble, unpublished data] suggesting some mechanism for higher gene flow than is currently appreciated.

Our data support the latter theory by indicating that there are only two populations across most of the range of the eastern box turtle. Furthermore the Appalachian Mountains may act or have acted as a barrier to gene flow at the continental scale, although eastern box turtles are currently known to inhabit all but the highest altitudes in North Carolina [19]. The finding that populations operate at large geographic scales is supported by previous work in the *Terrapene* species complex. In *T. ornata*, individuals from two sites 120 km apart were found to be genetically panmictic [66]. In *Terrapene c. carolina*, Marsack and Swanson [24] found that individuals separated by 30 to 70 km in southwestern Michigan also constituted a single population. Hagood [23] and Butler and colleagues [25] documented low genetic structure in *T. c. carolina* across larger distances of 160 km and 250 km, respectively. These studies suggest that the approach taken in this study, evaluating population genetic patterns at the scale of the species range, is the appropriate approach for the eastern box turtle.

Our data also indicate that isolation by distance is operating over relatively large spatial scales across the range of this species. The spatial autocorrelation analysis demonstrated that at approximately 300 – 500 km, mean pairwise genetic distances begin to exceed the average for the entire data set, suggesting a geographic extent at which populations operate. The same analysis by Hagood [23] in *T. c. carolina* returned a similar result of 450 to 650 km, which is approximately the distance from the Appalachian Mountains to the edge of the species range. These results, combined with the Geneland and Structure results, describe a species that though apparently highly philopatric, has (or had) high gene flow across vast areas. Though box turtles are reported throughout much of Appalachia [19], cryptic barriers to gene flow such as terrain and elevation may cause subtle population barriers in turtles [67] that may result in the Appalachians serving as a modest barrier.

Overall, we detected little evidence that habitat fragmentation is so far affecting population genetic structure in eastern box turtles. The shape and scope of the populations appear to be more consistent with a historical landscape than with current patterns of landscape fragmentation (Fig 1). Individuals from as far distant as eastern Tennessee and southwestern Michigan were assigned to the same population and few private alleles were detected from samples across these geographically distant populations (Table S1). The exception was possibly a signal of incipient decline: we did find evidence that migration between the two rangewide populations has recently been reduced or eliminated, a result expected in the presence of increasing habitat fragmentation. However, generation times can be long in *Terrapene*, with longevity in the wild at least 100 years (N. Karraker, unpublished data; [18]). As few as three generations could have passed since European settlers started clearing large swaths of forests in the range of the eastern box turtle [68], suggesting the idea that the genetic signatures we see in *Terrapene* box turtles may be largely historical. The loss of migration events between the two populations may be the first signal of reduced gene flow.

Furthermore, though we do not know what historical levels were, genetic diversity appears to remain high. Allelic richness and observed heterozygosity are high (Table S1). Long-lived species that have experienced demographic declines yet retain high genetic diversity include the Nile crocodile [69] and the harpy eagle [70]. However, while the former has experienced population growth under the protection of a CITES listing, the latter has not exhibited any such rebound. To date, all long-term demographic studies of *Terrapene* have documented declines [20]–[22], suggesting that box turtles require more active management to further prevent declines and extirpations.

This work represents the first rangewide study of population genetics patterns in the *Terrapene carolina* complex and provides insights into the ecology of a subspecies that appears to have only begun to exhibit the effects of habitat fragmentation within the last few generations. Indeed, our data indicate that genetic diversity remains high across the range of this *Terrapene subspecies*, yet the detection of historical gene flow and the lack of recent immigration events are signatures of recently reduced population connectivity. The loss of genetic diversity is a major threat to chelonian species worldwide [16], [17]. However, in long-lived species with overlapping generations, signatures of genetic loss may be masked for decades or even centuries [71]. Turtles are some of the longest-living vertebrates on the planet and yet many have suffered severe demographic declines, necessitating immediate management plans.

## Supporting Information

Table S1 **Rangewide population genetic parameter values for the eastern box turtle** ***Terrapene. c. carolina*** **.**
(PDF)

Table S2 **Locus-specific heterozygosities for populations of the eastern box turtle** ***Terrapene c. carolina*** **.**
(PDF)
