# Supplementary material for: Unexpectedly Low Rangewide Population Genetic Structure of the Imperiled Eastern Box Turtle Terrapene c. carolina
Source: PLoS One. 2014 Mar 19;9(3):e92274. doi: 10.1371/journal.pone.0092274 (PMC3960240; doi:10.1371/journal.pone.0092274)
Supplement: Table S1 — Rangewide population genetic parameter values for the eastern box turtle Terrapene. c. carolina . (PDF) [file pone.0092274.s001.pdf]

Table S1 Rangewide population genetic parameter values for the eastern box turtle, *Terrapene. c. carolina*, including sample size, mean allelic richness (N_A), mean inbreeding coefficient (F_{IS}), observed heterozygosity (H_O) and expected heterozygosity (H_E).

**Population**	**n**	**Mean N_A**	**Private alleles**	**F_{IS}**	**H_O**	**H_E**	**State legal status[3]**
**All samples***	799[1]	35.1	na	0.123	0.764	0.866	na
**Genetic populations**							
Western*	549[1]	32.6	39[2]	0.115	0.778	0.872	na
Eastern*	250[1]	21.3	13[2]	0.106	0.733	0.815	na
**Management populations**							
Southwest Michigan*	35	14.9	2	0.155	0.736	0.862	a
Lafayette, Indiana*	31	15.1	0	0.090	0.797	0.861	b
Sugar Creek, Indiana*	25	15.3	1	0.084	0.807	0.879	b
Hillenbrand Fish & Wildlife Area, Indiana*	44	17.6	1	0.101	0.782	0.864	b
Patoka River National Wildlife Refuge, Indiana*	69	19.1	4	0.078	0.808	0.864	b
Hovey Lake Fish & Wildlife Area, Indiana*	20	13.1	1	0.066	0.822	0.879	b
Hardwood Ecosystem Experiment, Indiana*	24	13.8	4	0.118	0.799	0.873	b
Blue River, Indiana*	74	20.6	6	0.116	0.776	0.871	b
Seymour, Indiana*	20	13.1	2	0.106	0.770	0.859	b
Big Oaks National Wildlife Refuge, Indiana*	31	14.8	0	0.116	0.771	0.868	b
Shawnee National Forest, Illinois*	29	14.1	2	0.104	0.778	0.861	c
Land Between the Lakes National Recreation Area, Kentucky*	36	17.0	2	0.088	0.801	0.877	c
Shawnee State Forest, Ohio*	30	13.5	2	0.080	0.789	0.857	d

Table S1 (cont.) Rangewide population genetic parameter valuess for the eastern box turtle, *Terrapene. c. carolina*, including mean allelic richness (N_A), mean inbreeding coefficient (F_{IS}), observed heterozygosity (H_O) and expected heterozygosity (H_E).

Population	n	Mean N_A	Private alleles	F_{IS}	H_O	H_E	State legal status[3]
Gettysburg National Military Park, Pennsylvania*	28	11.4	1	0.067	0.714	0.775	e
Long Island, New York*	25	11.5	0	0.080	0.741	0.808	a
Rock Creek Park, District of Columbia	9	7.6	0	0.073	0.665	0.794	f
Gaithersburg, Maryland	12	8.6	0	0.056	0.746	0.793	g
Jug Bay Wetlands Sanctuary, Maryland*	26	12.2	2	0.146	0.716	0.817	g
Muddy Branch Park, Maryland*	22	10.8	1	0.124	0.724	0.829	g
Wheaton Regional Park, Maryland*	39	11.8	1	0.131	0.678	0.780	g
Patuxent Wildlife Research Center, Maryland*	48	15.3	3	0.069	0.769	0.816	g
Isle of Wight Wildlife Management Area, Maryland*	10	7.6	2	0.111	0.734	0.820	g
Richmond, Virginia*	24	11.4	2	0.065	0.749	0.826	g
Oak Ridge, Tennessee*	24	13.2	12	0.135	0.718	0.840	g
Western North Carolina*	9	8.5	1	0.170	0.720	0.875	g
Chattahoochee National Forest, Georgia*	15	9.9	0	0.176	0.696	0.834	g

*Significantly out of Hardy-Weinberg equilibrium

[1]Total includes the rarefaction to n = 25 for both the deeply sampled Hardwood Ecosystem Experiment and Oak Ridge populations to reduce any effect of kinship.

[2]These private alleles are private only with respect to the other genetic population, not to the management populations.

[3]Codes: a = "Special concern", b = "State species of special concern", c = "Collection allowed, sale prohibited", d = "Species of concern", e = "No take", f = Species of greatest conservation need", g = "Nongame".
