# Supplementary material for: Unexpectedly Low Rangewide Population Genetic Structure of the Imperiled Eastern Box Turtle Terrapene c. carolina
Source: PLoS One. 2014 Mar 19;9(3):e92274. doi: 10.1371/journal.pone.0092274 (PMC3960240; doi:10.1371/journal.pone.0092274)
Supplement: Table S2 — Locus-specific heterozygosities for populations of the eastern box turtle Terrapene c. carolina . (PDF) [file pone.0092274.s002.pdf]

Table S2 Locus-specific heterozygosities for populations of the eastern box turtle *Terrapene c. carolina*. Allelic richnesses (N_A), inbreeding coefficients (F_{IS}), and observed (H_O) and expected (H_E) heterozygosities for all samples, both genetic populations, and all management units by locus. Populations with an * are significantly out of Hardy-Weinberg equilibrium.

Population	Locus	N_A	F_{IS}	H_O	H_E
**All samples (n=799)***	TCC_di_045	34	0.354	0.604	0.935
	TCC_di_082	30	0.078	0.804	0.872
	TCC_di_189	18	0.309	0.444	0.642
	TCC_di_300	53	0.018	0.908	0.925
	TCC_di_318	47	0.065	0.881	0.942
	TCC_di_345	83	0.208	0.747	0.943
	TCC_di_352	22	0.026	0.861	0.884
	TCC_di_366	16	0.038	0.789	0.821
	TCC_tetra_012/342	18	0.211	0.563	0.713
	TCC_tetra_043	36	0.019	0.9	0.917
	TCC_tetra_070	29	0.026	0.903	0.927
	**mean**	**35.1**	**0.123**	**0.764**	**0.866**
**Genetic populations**					
Western*	TCC_di_045	32	0.317	0.629	0.922
	TCC_di_082	30	0.094	0.795	0.877
	TCC_di_189	18	0.357	0.408	0.634
	TCC_di_300	48	0.024	0.906	0.929
	TCC_di_318	46	0.062	0.878	0.937
	TCC_di_345	73	0.16	0.793	0.945
	TCC_di_352	20	0.005	0.872	0.876
	TCC_di_366	13	0.046	0.786	0.824
	TCC_tetra_012/342	18	0.165	0.67	0.803
	TCC_tetra_043	35	0.006	0.91	0.916
	TCC_tetra_070	26	0.025	0.905	0.928
	**mean**	**32.6**	**0.115**	**0.778**	**0.872**
Eastern*	TCC_di_045	22	0.37	0.549	0.872
	TCC_di_082	14	0.027	0.822	0.844
	TCC_di_189	8	0.145	0.523	0.612
	TCC_di_300	35	-0.027	0.913	0.889
	TCC_di_318	29	0.049	0.887	0.933
	TCC_di_345	41	0.295	0.643	0.913
	TCC_di_352	16	0.013	0.839	0.851
	TCC_di_366	12	0.016	0.798	0.811
	TCC_tetra_012/342	13	0.209	0.319	0.404
	TCC_tetra_043	22	0.044	0.872	0.912
	TCC_tetra_070	22	0.025	0.9	0.923
	**mean**	**21.3**	**0.106**	**0.733**	**0.815**
**Management populations**					
Southwest Michigan*	TCC_di_045	16	0.408	0.543	0.918
	TCC_di_082	18	0.054	0.857	0.906
	TCC_di_189	6	0.544	0.313	0.685
	TCC_di_300	19	0.06	0.886	0.942
	TCC_di_318	25	0.07	0.857	0.922
	TCC_di_345	22	0.23	0.714	0.928
	TCC_di_352	9	-0.002	0.829	0.827
	TCC_di_366	6	0.025	0.771	0.792
	TCC_tetra_012/342	12	0.25	0.559	0.745
	TCC_tetra_043	16	0.066	0.848	0.909
	TCC_tetra_070	15	-0.001	0.914	0.913

Population	Locus	N_A	F_{IS}	H_O	H_E
	**mean**	**14.9**	**0.155**	**0.736**	**0.862**
Tippecanoe & Warren Counties, IN*	TCC_di_045	17	0.172	0.71	0.857
	TCC_di_082	17	0.169	0.767	0.922
	TCC_di_189	5	0.477	0.267	0.51
	TCC_di_300	22	0.041	0.903	0.941
	TCC_di_318	21	0.007	0.935	0.942
	TCC_di_345	19	0.028	0.903	0.929
	TCC_di_352	14	-0.011	0.903	0.894
	TCC_di_366	8	0.049	0.774	0.814
	TCC_tetra_012/342	13	0.069	0.8	0.859
	TCC_tetra_043	15	-0.126	1	0.888
	TCC_tetra_070	15	0.116	0.806	0.912
	**mean**	**15.1**	**0.09**	**0.797**	**0.861**
Parke County, IN*	TCC_di_045	16	0.162	0.76	0.907
	TCC_di_082	16	0.125	0.8	0.914
	TCC_di_189	4	0.187	0.435	0.535
	TCC_di_300	16	0.002	0.92	0.922
	TCC_di_318	25	-0.004	0.96	0.956
	TCC_di_345	22	0.167	0.8	0.961
	TCC_di_352	11	0.047	0.84	0.882
	TCC_di_366	10	-0.075	0.88	0.818
	TCC_tetra_012/342	14	0.212	0.72	0.914
	TCC_tetra_043	18	-0.04	0.96	0.923
	TCC_tetra_070	16	0.146	0.8	0.937
	**mean**	**15.3**	**0.084**	**0.807**	**0.879**
Hillenbrand Fish & Wildlife Area, IN*	TCC_di_045	18	0.327	0.591	0.878
	TCC_di_082	17	0.085	0.795	0.869
	TCC_di_189	5	0.269	0.455	0.622
	TCC_di_300	22	0.009	0.909	0.917
	TCC_di_318	27	0.062	0.886	0.945
	TCC_di_345	30	0.132	0.818	0.943
	TCC_di_352	13	0.015	0.841	0.854
	TCC_di_366	9	0.131	0.698	0.803
	TCC_tetra_012/342	16	0.075	0.786	0.849
	TCC_tetra_043	18	0.037	0.864	0.897
	TCC_tetra_070	19	-0.036	0.955	0.922
	**mean**	**17.6**	**0.101**	**0.782**	**0.864**
Patoka River National Wildlife Refuge, IN*	TCC_di_045	22	0.183	0.754	0.922
	TCC_di_082	16	0.083	0.783	0.854
	TCC_di_189	8	0.435	0.304	0.538
	TCC_di_300	24	-0.021	0.928	0.909
	TCC_di_318	28	-0.009	0.957	0.948
	TCC_di_345	34	0.121	0.841	0.956
	TCC_di_352	13	-0.063	0.913	0.859
	TCC_di_366	11	0.015	0.803	0.816
	TCC_tetra_012/342	16	0.151	0.725	0.853
	TCC_tetra_043	19	-0.044	0.957	0.916
	TCC_tetra_070	19	0.005	0.928	0.933
	**mean**	**19.1**	**0.078**	**0.808**	**0.864**
Hovey Lake Fish & Wildlife Area, IN*	TCC_di_045	15	0.244	0.7	0.926
	TCC_di_082	11	0.159	0.75	0.892
	TCC_di_189	8	0.143	0.6	0.7

Population	Locus	N_A	F_{IS}	H_O	H_E
	TCC_di_300	16	-0.018	0.95	0.933
	TCC_di_318	19	-0.001	0.95	0.949
	TCC_di_345	15	0.123	0.8	0.912
	TCC_di_352	11	0.079	0.8	0.868
	TCC_di_366	8	-0.061	0.85	0.801
	TCC_tetra_012/342	11	0.018	0.85	0.866
	TCC_tetra_043	15	0.075	0.842	0.911
	TCC_tetra_070	15	-0.037	0.95	0.916
	**mean**	**13.1**	**0.066**	**0.822**	**0.879**
Hardwood Ecosystem Experiment, IN*	TCC_di_045	13	0.223	0.7	0.901
	TCC_di_082	15	0.092	0.833	0.918
	TCC_di_189	6	0.384	0.391	0.635
	TCC_di_300	18	0.015	0.913	0.927
	TCC_di_318	20	0.028	0.917	0.943
	TCC_di_345	23	0.134	0.826	0.954
	TCC_di_352	9	0.18	0.708	0.864
	TCC_di_366	8	0.13	0.708	0.814
	TCC_tetra_012/342	11	0.177	0.652	0.792
	TCC_tetra_043	15	-0.033	0.957	0.926
	TCC_tetra_070	14	-0.034	0.958	0.927
	**mean**	**13.8**	**0.118**	**0.779**	**0.873**
Blue River, IN*	TCC_di_045	\	0.44	0.514	0.918
	TCC_di_082	23	0.044	0.838	0.876
	TCC_di_189	7	0.384	0.403	0.654
	TCC_di_300	29	-0.005	0.932	0.927
	TCC_di_318	32	0.047	0.903	0.947
	TCC_di_345	34	0.174	0.784	0.949
	TCC_di_352	15	-0.007	0.877	0.871
	TCC_di_366	12	0.094	0.75	0.828
	TCC_tetra_012/342	16	0.166	0.657	0.788
	TCC_tetra_043	20	-0.037	0.932	0.899
	TCC_tetra_070	18	-0.019	0.945	0.927
	**mean**	**20.6**	**0.116**	**0.776**	**0.871**
Seymour, IN*	TCC_di_045	14	0.358	0.6	0.934
	TCC_di_082	11	0.103	0.8	0.892
	TCC_di_189	4	0.214	0.444	0.565
	TCC_di_300	18	0.098	0.85	0.942
	TCC_di_318	19	0.101	0.842	0.937
	TCC_di_345	18	0.16	0.8	0.953
	TCC_di_352	16	-0.069	0.947	0.886
	TCC_di_366	8	-0.017	0.882	0.868
	TCC_tetra_012/342	8	0.096	0.556	0.614
	TCC_tetra_043	13	0.133	0.8	0.922
	TCC_tetra_070	15	-0.014	0.95	0.937
	**mean**	**13.1**	**0.106**	**0.77**	**0.859**
Big Oaks National Wildlife Refuge, IN*	TCC_di_045	14	0.468	0.484	0.909
	TCC_di_082	12	0.175	0.677	0.821
	TCC_di_189	6	0.38	0.419	0.677
	TCC_di_300	17	0.116	0.806	0.912
	TCC_di_318	24	0.058	0.871	0.924
	TCC_di_345	20	0.136	0.806	0.933
	TCC_di_352	14	-0.078	0.935	0.868

Population	Locus	N_A	F_{IS}	H_O	H_E
	TCC_di_366	9	-0.094	0.9	0.823
	TCC_tetra_012/342	15	0.137	0.71	0.822
	TCC_tetra_043	17	-0.041	0.968	0.93
	TCC_tetra_070	15	0.023	0.903	0.924
	**mean**	**14.8**	**0.116**	**0.771**	**0.868**
Shawnee National Forest, IL*	TCC_di_045	15	0.331	0.607	0.908
	TCC_di_082	15	0.021	0.862	0.881
	TCC_di_189	6	0.385	0.345	0.56
	TCC_di_300	17	-0.056	0.966	0.914
	TCC_di_318	22	0.09	0.862	0.948
	TCC_di_345	19	0.203	0.724	0.909
	TCC_di_352	10	0.007	0.828	0.833
	TCC_di_366	11	-0.044	0.893	0.855
	TCC_tetra_012/342	11	0.042	0.793	0.828
	TCC_tetra_043	14	0.099	0.821	0.912
	TCC_tetra_070	15	0.07	0.857	0.922
	**mean**	**14.1**	**0.104**	**0.778**	**0.861**
Land Between the Lakes National Recreation Area, KY*	TCC_di_045	21	0.206	0.75	0.944
	TCC_di_082	15	0.071	0.833	0.897
	TCC_di_189	7	0.124	0.556	0.634
	TCC_di_300	24	0.077	0.861	0.933
	TCC_di_318	24	0.035	0.917	0.95
	TCC_di_345	27	0.064	0.889	0.95
	TCC_di_352	13	0.008	0.861	0.868
	TCC_di_366	10	-0.011	0.806	0.797
	TCC_tetra_012/342	14	0.321	0.583	0.86
	TCC_tetra_043	14	0.067	0.833	0.893
	TCC_tetra_070	18	0.009	0.917	0.925
	**mean**	**17**	**0.088**	**0.801**	**0.877**
Shawnee State Forest, OH*	TCC_di_045	13	0.198	0.72	0.898
	TCC_di_082	11	0.042	0.833	0.87
	TCC_di_189	7	0.299	0.5	0.713
	TCC_di_300	17	-0.004	0.933	0.929
	TCC_di_318	16	-0.012	0.897	0.886
	TCC_di_345	18	0.142	0.793	0.924
	TCC_di_352	12	0.124	0.767	0.875
	TCC_di_366	7	0.054	0.778	0.822
	TCC_tetra_012/342	13	-0.086	0.733	0.675
	TCC_tetra_043	18	0.028	0.889	0.915
	TCC_tetra_070	16	0.09	0.833	0.916
	**mean**	**13.5**	**0.08**	**0.789**	**0.857**
Gettysburg National Military Park, PA*	TCC_di_045	13	0.263	0.63	0.855
	TCC_di_082	9	0.04	0.815	0.849
	TCC_di_189	4	-0.002	0.536	0.534
	TCC_di_300	16	-0.107	1	0.903
	TCC_di_318	17	0.148	0.786	0.922
	TCC_di_345	17	0.266	0.667	0.908
	TCC_di_352	9	0.049	0.786	0.826
	TCC_di_366	8	0.071	0.75	0.808
	TCC_tetra_012/342	3	-0.025	0.107	0.104
	TCC_tetra_043	15	0.071	0.85	0.914
	TCC_tetra_070	14	-0.032	0.929	0.899

Population	Locus	N_A	F_{IS}	H_O	H_E
	**mean**	**11.4**	**0.067**	**0.714**	**0.775**
Long Island, NY*	TCC_di_045	8	0.528	0.4	0.847
	TCC_di_082	11	0.051	0.8	0.843
	TCC_di_189	5	-0.145	0.64	0.559
	TCC_di_300	12	-0.01	0.833	0.825
	TCC_di_318	17	0.057	0.875	0.928
	TCC_di_345	17	0.247	0.696	0.924
	TCC_di_352	11	-0.035	0.84	0.812
	TCC_di_366	7	0.036	0.792	0.822
	TCC_tetra_012/342	9	0.214	0.4	0.509
	TCC_tetra_043	14	-0.065	0.96	0.902
	TCC_tetra_070	15	-0.002	0.92	0.918
	**mean**	**11.5**	**0.08**	**0.741**	**0.808**
Rock Creek Park, DC	TCC_di_045	11	0.132	0.778	0.896
	TCC_di_082	8	0.339	0.556	0.84
	TCC_di_189	4	0.169	0.444	0.535
	TCC_di_300	9	0.02	0.875	0.893
	TCC_di_318	12	-0.067	1	0.938
	TCC_di_345	10	0.284	0.667	0.931
	TCC_di_352	8	-0.2	1	0.833
	TCC_di_366	6	0.067	0.778	0.833
	TCC_tetra_012/342	3	-0.053	0.333	0.317
	TCC_tetra_043	1	NA	0	NA
	TCC_tetra_070	11	0.038	0.889	0.924
	**mean**	**7.55**	**0.073**	**0.665**	**0.794**
Gaithersburg, MD	TCC_di_045	9	0.225	0.667	0.86
	TCC_di_082	7	-0.005	0.833	0.83
	TCC_di_189	4	0.12	0.417	0.473
	TCC_di_300	9	-0.052	0.917	0.871
	TCC_di_318	14	0.012	0.917	0.928
	TCC_di_345	9	0.25	0.667	0.889
	TCC_di_352	11	0.016	0.917	0.932
	TCC_di_366	6	0.2	0.667	0.833
	TCC_tetra_012/342	4	-0.059	0.3	0.283
	TCC_tetra_043	10	-0.099	1	0.91
	TCC_tetra_070	11	0.01	0.909	0.918
	**mean**	**8.55**	**0.056**	**0.746**	**0.793**
Jug Bay Wetlands Sanctuary, MD*	TCC_di_045	18	0.21	0.731	0.925
	TCC_di_082	10	0.223	0.654	0.842
	TCC_di_189	6	0.401	0.385	0.642
	TCC_di_300	12	-0.011	0.88	0.87
	TCC_di_318	18	0.051	0.885	0.932
	TCC_di_345	20	0.307	0.654	0.944
	TCC_di_352	9	-0.018	0.885	0.869
	TCC_di_366	7	-0.053	0.84	0.798
	TCC_tetra_012/342	6	0.443	0.192	0.345
	TCC_tetra_043	15	0.035	0.885	0.917
	TCC_tetra_070	13	0.015	0.885	0.898
	**mean**	**12.2**	**0.146**	**0.716**	**0.817**
Muddy Branch Park, MD*	TCC_di_045	10	0.503	0.455	0.915
	TCC_di_082	10	-0.17	1	0.855
	TCC_di_189	5	0	0.6	0.6

Population	Locus	N_A	F_{IS}	H_O	H_E
	TCC_di_300	14	-0.004	0.905	0.901
	TCC_di_318	17	0.073	0.864	0.932
	TCC_di_345	14	0.565	0.409	0.939
	TCC_di_352	10	0.094	0.727	0.803
	TCC_di_366	8	-0.019	0.864	0.847
	TCC_tetra_012/342	6	0.256	0.381	0.512
	TCC_tetra_043	10	0.064	0.846	0.904
	TCC_tetra_070	15	-0.001	0.909	0.908
	**mean**	**10.8**	**0.124**	**0.724**	**0.829**
Wheaton Regional Park, MD*	TCC_di_045	12	0.55	0.368	0.818
	TCC_di_082	11	-0.032	0.795	0.77
	TCC_di_189	6	0.363	0.378	0.594
	TCC_di_300	15	-0.068	0.921	0.862
	TCC_di_318	21	-0.013	0.949	0.937
	TCC_di_345	18	0.188	0.703	0.865
	TCC_di_352	11	0.046	0.821	0.86
	TCC_di_366	9	-0.03	0.821	0.797
	TCC_tetra_012/342	6	0.025	0.257	0.264
	TCC_tetra_043	8	0.36	0.571	0.893
	TCC_tetra_070	13	0.056	0.872	0.923
	**mean**	**11.8**	**0.131**	**0.678**	**0.78**
Patuxent Wildlife Research Center, MD*	TCC_di_045	15	0.142	0.689	0.803
	TCC_di_082	12	-0.008	0.875	0.868
	TCC_di_189	6	0.224	0.478	0.616
	TCC_di_300	23	-0.008	0.917	0.909
	TCC_di_318	21	0.042	0.875	0.913
	TCC_di_345	26	0.241	0.688	0.906
	TCC_di_352	12	-0.074	0.915	0.852
	TCC_di_366	10	-0.018	0.83	0.815
	TCC_tetra_012/342	9	0.229	0.34	0.442
	TCC_tetra_043	18	0.013	0.915	0.927
	TCC_tetra_070	16	-0.019	0.938	0.92
	**mean**	**15.3**	**0.069**	**0.769**	**0.816**
Isle of Wight Wildlife Management Area, MD*	TCC_di_045	7	0.723	0.2	0.722
	TCC_di_082	9	0.006	0.9	0.906
	TCC_di_189	7	0.111	0.778	0.875
	TCC_di_300	9	-0.006	0.9	0.894
	TCC_di_318	9	-0.013	0.9	0.889
	TCC_di_345	9	0.154	0.7	0.828
	TCC_di_352	5	-0.125	0.8	0.711
	TCC_di_366	5	-0.043	0.8	0.767
	TCC_tetra_012/342	7	0.194	0.6	0.744
	TCC_tetra_043	8	0.187	0.7	0.861
	TCC_tetra_070	9	0.034	0.8	0.828
	**mean**	**7.64**	**0.111**	**0.734**	**0.82**
Richmond, VA*	TCC_di_045	13	0.36	0.583	0.911
	TCC_di_082	12	-0.088	0.913	0.839
	TCC_di_189	5	0.017	0.625	0.636
	TCC_di_300	14	-0.088	0.955	0.878
	TCC_di_318	16	-0.064	0.958	0.9
	TCC_di_345	11	0.455	0.478	0.877
	TCC_di_352	10	0.121	0.75	0.853

Population	Locus	N_A	F_{IS}	H_O	H_E
	TCC_di_366	6	0.062	0.75	0.8
	TCC_tetra_012/342	8	0.118	0.478	0.542
	TCC_tetra_043	17	0.05	0.875	0.921
	TCC_tetra_070	13	0.054	0.875	0.925
	**mean**	**11.4**	**0.091**	**0.749**	**0.826**
Knoxville, TN*	TCC_di_045	17	0.503	0.458	0.922
	TCC_di_082	12	0.135	0.75	0.867
	TCC_di_189	7	0.21	0.609	0.771
	TCC_di_300	15	-0.015	0.917	0.903
	TCC_di_318	16	0.22	0.667	0.855
	TCC_di_345	20	0.213	0.75	0.953
	TCC_di_352	14	0.085	0.833	0.91
	TCC_di_366	9	0.025	0.818	0.839
	TCC_tetra_012/342	7	-0.045	0.435	0.416
	TCC_tetra_043	16	0.119	0.792	0.899
	TCC_tetra_070	12	0.039	0.875	0.91
	**mean**	**13.2**	**0.135**	**0.718**	**0.84**
Western North Carolina*	TCC_di_045	10	0.486	0.5	0.973
	TCC_di_082	7	0.238	0.667	0.875
	TCC_di_189	4	-0.167	0.778	0.667
	TCC_di_300	11	0.045	0.889	0.931
	TCC_di_318	9	0.35	0.556	0.854
	TCC_di_345	10	0.164	0.778	0.931
	TCC_di_352	11	0.182	0.778	0.951
	TCC_di_366	8	0.118	0.778	0.882
	TCC_tetra_012/342	6	0.252	0.556	0.743
	TCC_tetra_043	5	0.143	0.75	0.875
	TCC_tetra_070	12	0.059	0.889	0.944
	**mean**	**8.45**	**0.17**	**0.72**	**0.875**
Chattahoochee National Forest, GA*	TCC_di_045	10	0.495	0.462	0.913
	TCC_di_082	11	0.202	0.667	0.836
	TCC_di_189	5	0.158	0.571	0.679
	TCC_di_300	14	0	0.923	0.923
	TCC_di_318	15	0.138	0.8	0.929
	TCC_di_345	12	0.228	0.714	0.926
	TCC_di_352	7	-0.114	0.933	0.838
	TCC_di_366	6	0.27	0.6	0.821
	TCC_tetra_012/342	5	0.456	0.267	0.49
	TCC_tetra_043	12	0.043	0.857	0.896
	TCC_tetra_070	12	0.062	0.867	0.924
	**mean**	**9.91**	**0.176**	**0.696**	**0.834**
